# New developments in canine hepatozoonosis in North America: a review

**DOI:** 10.1186/1756-3305-2-S1-S5

**Published:** 2009-03-26

**Authors:** Susan E Little, Kelly E Allen, Eileen M Johnson, Roger J Panciera, Mason V Reichard, Sidney A Ewing

**Affiliations:** 1 Department of Veterinary Pathobiology, Center for Veterinary Health Sciences, Oklahoma State University, Stillwater, OK, USA

## Abstract

Canine hepatozoonosis is caused by *Hepatozoon canis *and *Hepatozoon americanum*, apicomplexan parasites transmitted to dogs by ingestion of infectious stages. Although the two agents are phylogenetically related, specific aspects, including characteristics of clinical disease and the natural history of the parasites themselves, differ between the two species. Until recently, *H. canis *infections had not been clearly documented in North America, and autochthonous infection with *H. americanum *has yet to be reported outside of the southern United States. However, recent reports demonstrate *H. canis *is present in areas of North America where its vector tick, *Rhipicephalus sanguineus*, has long been endemic, and that the range of *H. americanum *is likely expanding along with that of its vector tick, *Amblyomma maculatum*; co-infections with the two organisms have also been identified. Significant intraspecific variation has been reported in the 18S rRNA gene sequence of both *Hepatozoon *spp.-infecting dogs, suggesting that each species may represent a complex of related genogroups rather than well-defined species. Transmission of *H. americanum *to dogs via ingestion of cystozoites in muscle of infected vertebrates was recently demonstrated, supporting the concept of predation as a means of natural transmission. Although several exciting advances have occurred in recent years, much remains to be learned about patterns of infection and the nature of clinical disease caused by the agents of canine hepatozoonosis in North America.

## Background

*Hepatozoon canis *has long been recognised to infect and cause disease in dogs in Asia, southern Europe, the Middle East, Africa and South America. In 1978, cases of hepatozoonosis were recognised for the first time in the southern United States and initially attributed to a more pathogenic strain of *H. canis *[1]. However, subsequent work demonstrated that the causative agent was a distinct species, *H. americanum*, and the disease induced became known as American canine hepatozoonosis [2-6]. The life cycle of both *H. canis *and *H. americanum *involves a tick-definitive host, where fertilisation, oocyst formation and sporogony occur, and a canine or other mammalian intermediate host, in which merogony and gamont formation occurs [7]. Gamonts are acquired by ticks during feeding, but sporozoites are released to infect the intermediate host only upon ingestion of the infected tick itself [8]. Although somewhat similar in overall life cycle, the two *Hepatozoon *spp. reported from dogs differ in several ways, including aspects of the clinical disease they cause in dogs, the species of tick used as a definitive host and their geographic distribution.

## Clinical features of canine hepatozoonoses

Dogs with American canine hepatozoonosis present with severe, febrile disease characterised by lethargy, myalgia, lameness and mucopurulent ocular discharge. Radiographs may reveal periosteal proliferation of long bones. A profound neutrophilic leukocytosis is often present, but gamonts are only rarely found in blood smears [9]. In contrast, dogs with canine hepatozoonosis caused by *H. canis *are often clinically normal or develop only mild disease. With high parasitemia or in cases of co-infection with other tick-borne disease agents, dogs with *H. canis *may develop mild to severe clinical disease characterised by fever, lethargy and emaciation. Osteoproliferative lesions are only rarely described with *H. canis *infection [10], and thus radiographs are usually unremarkable. Leukocyte counts are also usually normal or only slightly elevated, and gamonts are numerous, infecting as many as 100% of neutrophils observed [7].

## Tick vectors and geographic distribution of agents

### Hepatozoon americanum

The definitive host and tick vector of *H. americanum *is *Amblyomma maculatum*, the Gulf Coast tick, which in North America was historically limited to areas along the Gulf Coast and southern Atlantic coast [11]. This tick has also been reported from Central America and northern South America [12]. Reports of *H. americanum *infection in dogs outside of the U.S. are lacking, although one survey of wildlife described an 18S rDNA sequence 97% similar to that of *H. americanum *from a crab-eating fox from Brazil [13]. In recent decades, the range of *A. maculatum *has expanded northward with populations now established in areas of Oklahoma, Kansas, Kentucky and some other states [12,14]. In addition, *H. americanum *has been reported from California, Washington and Vermont [15], states outside the recognised range of the tick vector, presumably due to relocation of infected dogs from endemic areas.

### Hepatozoon canis

The main vector of *H. canis *is *Rhipicephalus sanguineus*, the brown dog tick, although other ticks have also been reported as hosts [16-20]. *Rhipicephalus sanguineus *is found in temperate and tropical regions worldwide, and cases of *H. canis *have been reported from southern Europe, Asia, Africa, the Middle East and South America [7]. However, prior to 2008, *H. canis *had not been definitively identified in dogs or wild canids in North America. Since that time, *H. canis *has been confirmed in 30 dogs in the United States by PCR, 14 of which were also infected with *H. americanum *[15,21], and identified in 5 dogs on the Caribbean island of Grenada [22]. Gamonts, presumably *H. canis*, were also identified on blood smear from one dog from the U.S. that had been PCR-confirmed as infected with *H. canis*, but not *H. americanum *[15]. Infection has also been identified by sequence-confirmed PCR in a grey fox from Georgia (M. Yabsley, pers. comm.), further supporting the interpretation that this organism is present and actively transmitted to canids in North America.

## Intraspecific variation

The only *Hepatozoon *species described from dogs to date are *H. canis *and *H. americanum*; both have variation in reported 18S rRNA gene sequences [21,23]. Although complete sequence data is available from only 2 dogs, identity among the 18S rDNA fragments reported from dogs infected with *H. americanum *ranges from 92.7–99.6% [21]. Among *H. canis *sequences, the identity reported is 97–100% [21,23]. Although strain variations likely account for the differences in sequence of 18S rRNA gene, the genogroup clusters from the sequence data available to date do not appear to correspond with geographic location of the dogs or severity of disease seen (data not shown). Acquisition of larger data sets with detailed information on clinical and travel history of the dogs will be necessary to more fully understand the significance, if any, of the sequence variation seen.

## Novel transmission routes

In both the *H. canis *and *H. americanum *systems, dogs have been shown capable of infecting immature ticks with gamonts that develop to infectious oocysts and are able to infect a new dog upon ingestion [24]. For *H. canis*, dogs are likely the preeminent reservoir host because the preferred vector tick, *R. sanguineus*, feeds preferentially on dogs in all three active life stages [25]. A variety of species of fox have also been reported infected with *H. canis *[13], and thus wild canids may play a role in maintaining a source of infection in nature. In contrast, immature *A. maculatum *are more frequently found feeding on ground-dwelling birds, rodents and rabbits than on canids, although all three stages have been reported from coyotes [11]. Infection of wild coyotes with *H. americanum *in *A. maculatum*-endemic areas has been documented [26,27]. Coyotes have been shown to be experimentally susceptible to infection and disease and are capable of infecting immature ticks [27,28]. However, the predominance of immature *A. maculatum *on other vertebrate species led to persistent questions about the role hosts other than canids may play in creating a source of infection for ticks [6,9].

Results from recent experiments confirm the validity of these suspicions. Ingestion of *H. americanum *sporozoites was shown to lead to the development of cystozoites in muscle tissue of cotton rats [29]. Muscle from infected rats was infectious to a dog, inducing the characteristic clinical disease of American canine hepatozoonosis [30]. Similar experiments with *H. americanum *in the past using muscle from infected dogs failed to result in transmission [2,31], and although monozoic cysts have been described in dogs infected with *H. canis *[32], to our knowledge no feeding experiments to evaluate their infectivity have been reported. Despite testing a number of animals in endemic areas, naturally occurring infection with *H. americanum *in wild rodents, rabbits or vertebrates other than canids has not yet been demonstrated ([33]; K. Allen, unpub. data), but evidence is mounting to support the role of wild vertebrates as an important paratenic host for this parasite. Transplacental infection has also been shown to occur with *H. canis *[34], but this transmission route has not been seen in *H. americanum*.

## Conclusion

Although these new data are exciting, many important questions remain about canine hepatozoonosis in North America. The variation in 18S rDNA sequence reported from infected dogs suggests that multiple strains or species of *Hepatozoon*, which could vary in their pathogenicity and life history patterns, may be infecting and causing disease in dogs [21]. Discovery of an infectious cystozoite stage in rodents suggests vertebrates other than canids could prove to be important in maintaining *H. americanum *infections in nature by serving as paratenic hosts that infect dogs through predation [29,30]; the ability of other vertebrates to infect immature ticks remains to be determined, as does the role, if any, of the monozoic cyst form described in dogs with *H. canis *[32] in transmitting infection. In addition, defining the extent to which *H. canis *infects dogs in North America is of great interest. In contrast to those caused by *H. americanum*, *H. canis *infections are often clinically inapparent, particularly in otherwise healthy dogs [7], and thus this agent may be more common in dogs in this region than currently realised. The presence of a number of other endemic tick-borne infections, including anaplasmosis, babesiosis, borreliosis, ehrlichiosis and Rocky Mountain spotted fever [35-37], provides ample opportunity for co-infection and more severe disease. The agents of hepatozoonosis, like those of other vector-borne diseases, present international challenges; these important questions can be addressed only through sustained collaboration between veterinarians and parasitologists worldwide with expertise in *H. canis *and *H. americanum*.

## Competing interests

Dr. Little (SEL) receives financial support for her research on ticks and tick-borne diseases from the veterinary pharmaceutical industry, the National Institutes of Health (USA) and private foundations.

## Authors' contributions

This work reviews recent findings from a collaborative effort among researchers at Oklahoma State University that identify and characterise *Hepatozoon *sp. infections in dogs and other animals with molecular techniques (SEL, KEA) and classical studies to describe lesions and parasite life stages in vertebrate and invertebrate hosts and identify the role of wild vertebrates in the maintenance and transmission of *H. americanum *(EMJ, MVR, RJP, SAE).

## References

[B1] Craig TM, Smallwood JE, Knauer KW, McGrath JP (1978). *Hepatozoon canis *infection in dogs: clinical, radiographic and hematological findings. JAVMA.

[B2] Vincent-Johnson NA, MacIntire DK, Lindsay DS, Lenz SD, Baneth G, Shkap V, Blagburn BL (1997). A new *Hepatozoon *species from dogs: description of the causative agent of canine hepatozoonosis in North America. J Parasitol.

[B3] Mathew JS, Bussche RA Van Den, Ewing SA, Malayer JR, Latha BR, Panciera RJ (2000). Phylogenetic relationships of *Hepatozoon *(Apicomplexa: Adeleorina) based on molecular, morphologic, and life-cycle characters. J Parasitol.

[B4] Panciera RJ, Mathew JS, Cummings CA, Duffy JC, Ewing SA, Kocan AA (2001). Comparison of tissue stages of *Hepatozoon americanum *in the dog using immunohistochemical and routine histologic methods. Vet Pathol.

[B5] Ewing SA, Mathew JS, Panciera RJ (2002). Transmission of *Hepatozoon americanum *(Apicomplexa: Adeleorina) by Ixodids (Acari: Ixodidae). J Med Entomol.

[B6] Ewing SA, Panciera RJ (2003). American canine hepatozoonosis. Clin Microbiol Rev.

[B7] Baneth G, Vincent-Johnson N, Shaw SE, Day MJ (2005). Hepatozoonosis. Arthorpod-borne infectious diseases of the dog and cat.

[B8] Baneth G, Samish M, Shkap V (2007). Life cycle of *Hepatozoon canis *(Apicomplexa: Adeleorina: Hepatozoidae) in the tick *Rhipicephalus sanguineus *and domestic dog (*Canis familiaris*). J Parasitol.

[B9] Panciera RJ, Ewing SA (2003). American canine hepatozoonosis. Anim Health Res Rev.

[B10] Marchetti V, Lubas G, Baneth G, Modenato M, Mancianti F (2009). Hepatozoonosis in a dog with skeletal involvement and meningoencephalomyelitis. Vet Clin Pathol.

[B11] Williams HR (2002). The biology and zoogeography of the Gulf Coast tick, *Amblyomma maculatum*, the potential vector of *Ehrlichia ruminantum *in the United States. Texas A&M Dissertation.

[B12] Estrada-Pena A, Venzal JM, Mangold AJ, Cafrune MM, Guglielmone AA (2005). The *Amblyomma maculatum *tick group: diagnostic characters, description of the larva of *A. parvitarsum*, 16S rDNA sequences, distribution, and hosts. Syst Parasitol.

[B13] Criado-Fornelio A, Ruas JL, Casado N, Farias NAR, Soares MP, Müller G, Brum JGW, Berne MEA, Buling-Saraña A, Barba-Carretero JC (2006). New molecular data on mammalian *Hepatozoon *species (Apicomplexa: Adeleorina) from Brazil and Spain. J Parasitol.

[B14] Sumner JW, Durden LA, Goddard J, Stromdahl EY, Clark KL, Reeves RK, Paddock CD (2007). Gulf Coast ticks (*Amblyomma maculatum*) and *Rickettsia parkeri*, United States. EID.

[B15] Li Y, Wang C, Allen K, Little S, Ahluwalia S, Gao D, Kaltenboeck B (2008). Diagnosis of canine *Hepatozoon *spp. infection by quantitative PCR. Vet Parasit.

[B16] Baneth G, Shkap V, Presentey BZ, Pipano E (1996). *Hepatozoon canis*: the prevalence of antibodies and gametocytes in dogs in Israel. Vet Res Commun.

[B17] McCully RM, Basson PA, Bigalke RD, DeVos V, Young E (1975). Observations on naturally acquired hepatozoonosis of wild carnivores and dogs in the Republic of South Africa. Onderstepoort J Vet Sci.

[B18] Murata T, Inoue M, Taura Y, Nakama S, Abe H, Fujisaki K (1995). Detection of *Hepatozoon canis *oocysts from ticks collected from the infected dogs. J Vet Med Sci.

[B19] Craig TM, Greene CE (1998). Hepatozoonosis. Infectious Diseases of the Dog and Cat.

[B20] O'Dwyer LH, Massard CL, Pereira de Souza JC (2001). *Hepatozoon canis *infection associated with dog ticks of rural areas of Rio de Janeiro State, Brazil. Vet Parasit.

[B21] Allen KE, Li Y, Kaltenboeck B, Johnson EM, Reichard MV, Panciera RJ, Little SE (2008). Diversity of *Hepatozoon *species in naturally infected dogs in the southern United States. Vet Parasitol.

[B22] Yabsley MJ, McKibben J, Macpherson CN, Cattan PF, Cherry NA, Hegarty BC, Breitschwerdt EB, O'Connor T, Chandrashekar R, Paterson T, Perea ML, Ball G, Friesen S, Goedde J, Henderson B, Sylvester W (2008). Prevalence of *Ehrlichia canis*, *Anaplasma platys*, *Babesia canis vogeli*, *Hepatozoon canis*, *Bartonella vinsonii berkhoffii*, and *Rickettsia *spp. in dogs from Grenada. Vet Parasitol.

[B23] Karagenc TI, Pasa S, Kirli G, Hosgor M, Bilgic HB, Ozon YH, Atasoy A, Eren H (2006). A parasitological, molecular and serological survey of *Hepatozoon canis *infection in dogs around the Aegean coast of Turkey. Vet Parasitol.

[B24] Mathew JS, Ewing SA, Panciera RJ, Woods JP (1998). Experimental transmission of *Hepatozoon americanum *Vincent-Johnson *et al*., 1997 to dogs by the Gulf Coast tick, *Amblyomma maculatum *Koch. Vet Parasitol.

[B25] Dantas-Torres F (2008). The brown dog tick, *Rhipicephalus sanguineus *(Latreille, 1806) (Acari: Ixodidae): from taxonomy to control. Vet Parasitol.

[B26] Kocan AA, Breshears M, Panciera RJ, Ewing SA, Barker RW (1999). Naturally occurring hepatozoonosis in coyotes from Oklahoma. J Wild Dis.

[B27] Kocan AA, Cummings CA, Panciera RJ, Mathew JS, Ewing SA, Barker RW (2000). Naturally occurring and experimentally transmitted *Hepatozoon americanum *in coyotes from Oklahoma. J Wild Dis.

[B28] Garrett JJ, Kocan AA, Reichard MV, Panciera RJ, Bahr RJ, Ewing SA (2005). Experimental infection of adult and juvenile coyotes with domestic dog and wild coyote isolates of *Hepatozoon americanum *(Apicomplexa:Adeleorina). J Wild Dis.

[B29] Johnson EM, Allen KE, Breshears MA, Panciera RJ, Little SE, Ewing SA (2007). Experimental transmission of *Hepatozoon americanum *to rodents. Vet Parasit.

[B30] Johnson EM, Allen KE, Panciera RJ, Little SE, Ewing SA (2008). Infectivity of *Hepatozoon americanum *cystozoites for a dog. Vet Parasitol.

[B31] Nordgren RM, Craig TM (1984). Experimental transmission of the Texas strain of *Hepatozoon canis*. Vet Parasitol.

[B32] Baneth G, Shkap V (2003). Monozoic cysts of *Hepatozoon canis*. J Parasitol.

[B33] Johnson EM, Allen KE, Panciera RJ, Ewing SA, Little SE, Reichard MV (2007). Field survey of rodents for *Hepatozoon *infections in an endemic focus of American canine hepatozoonosis. Vet Parasit.

[B34] Murata T, Inoue M, Tateyama S, Taura Y, Nakama S (1993). Vertical transmission of *Hepatozoon canis *in dogs. J Vet Med Sci.

[B35] Dantas-Torres F (2007). Rocky Mountain spotted fever. Lancet Infect Dis.

[B36] Bowman D, Little SE, Lorentzen L, Shields J, Sullivan MP, Carlin EP (2009). Prevalence and geographic distribution of *Dirofilaria immitis*, *Borrelia burgdorferi*, *Ehrlichia canis*, and *Anaplasma phagocytophilum *in dogs in the United States: results of a national clinic-based serologic survey. Vet Parasitol.

[B37] Yeagley TJ, Reichard MV, Hempstead JE, Allen KE, Parsons LM, White MA, Little SE, Meinkoth JH (2009). Detection of *Babesia gibsoni *and the canine small *Babesia *sp. "Spanish Isolate" in confiscated pit bull terriers. JAVMA.

